# Supplementary material for: Signals of complexity and fragmentation in accelerometer data
Source: PLoS One. 2025 Jul 9;20(7):e0326522. doi: 10.1371/journal.pone.0326522 (PMC12240345; doi:10.1371/journal.pone.0326522)
Supplement: S1 Text — (PDF) [file pone.0326522.s001.pdf]

# Signals of complexity and fragmentation in accelerometer data - Supplementary materials

Els Weinans, Jerrald L. Rector, Sarah Charman, Renae J. Stefanetti, Cecilia Jimenez-Moreno, Gráinne S. Gorman, Ingrid van de Leemput, Daniël van As, René Melis, & Baziel van Engelen

## 1 correlation between indicators

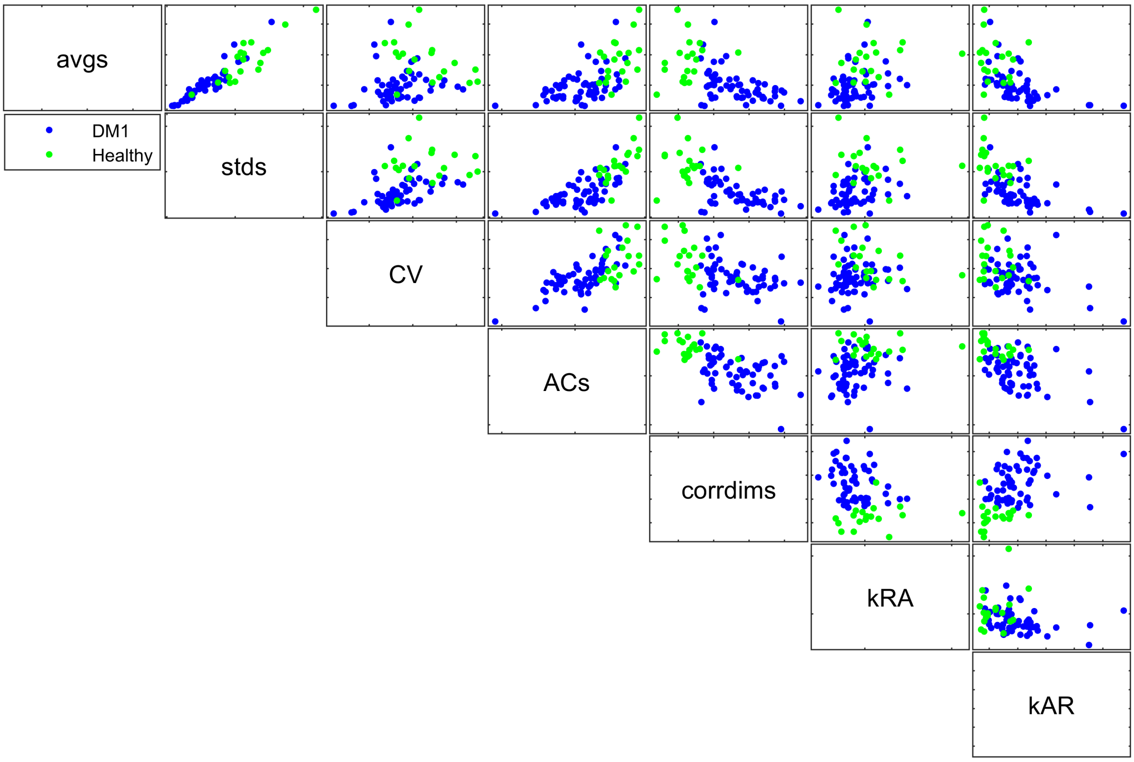


Figure 1: Visualization of Table 1 in main text: correlations between the different indicators of the activity of the signal (average, standard deviation, coefficient of variation, autocorrelation, correlation dimension, probability of rest after activity, and probability of activity after rest.

## 2 Power analysis

Since this was a highly exploratory study, we did not have a priori expectations for our effect size and thus could not perform a power analysis upfront. However, we did perform a power analysis a posteriori, with our found sample for the correlation dimension.

We took 1000 samples of a certain size both from our healthy group and our DM1 group. Next, we ran our analysis as normal. We calculated the power and the percentage of samples that led to a significant (p¡0.05) difference between the DM1 group and the healthy group. The power analysis indicates that for a sample size above 6, the difference between the two groups can be picked up (i.e. the power is above 0.95). Our sample size is well above this number.

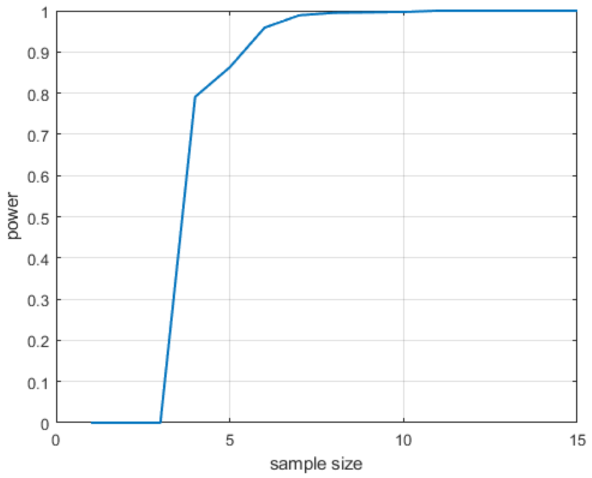


Figure 2: We took 1000 samples of different sizes for both the DM1 and the healthy group. For each sample size, we calculated how many of the 1000 samples would find a significant difference between the DM1 and the healthy group. For samples with a size of 6 of higher, the power is higher than 0.95, meaning that the sample size is sufficient to pick up on the difference between the groups.

## 3 Analysis with matched sample

Since our samples were unequal with respect to age and gender, we repeated the analysis using a subset of our 19 individuals from our DM1 group, which were chosen to match the healthy group with respect to age and gender. In this new sample, the DM1 group had an average age of 36.91 years (compared to 35.52 years in the healthy group) and both groups contained 42% males. Mann-Whitney U tests revealed that in this smaller sample, differences for the correlation dimension and kAR remained statistically significant (U=538, p<0.001 and U=480, p<0.01 respectively) and kRA is no longer significant (U=309, p=0.07). The data is represented in Fig.3.

Logistic regression models based on this reduced dataset show exploding confidence intervals. The lag-1 autocorrelation is the only variable that had a statistical significant effect in model 1 (table 1). However, based on both AIC and BIC, the inclusion of correlation dimension (model 2) is more likely than the model with only traditional analysis (model 1). The addition of kAR (model 3) or kRA (model 4) do not improve the model sufficiently.

The explosion in confidence intervals for model 2 suggests that this reduced dataset is underpowered for the analysis and therefore we cannot draw any strong conclusions from this analysis.

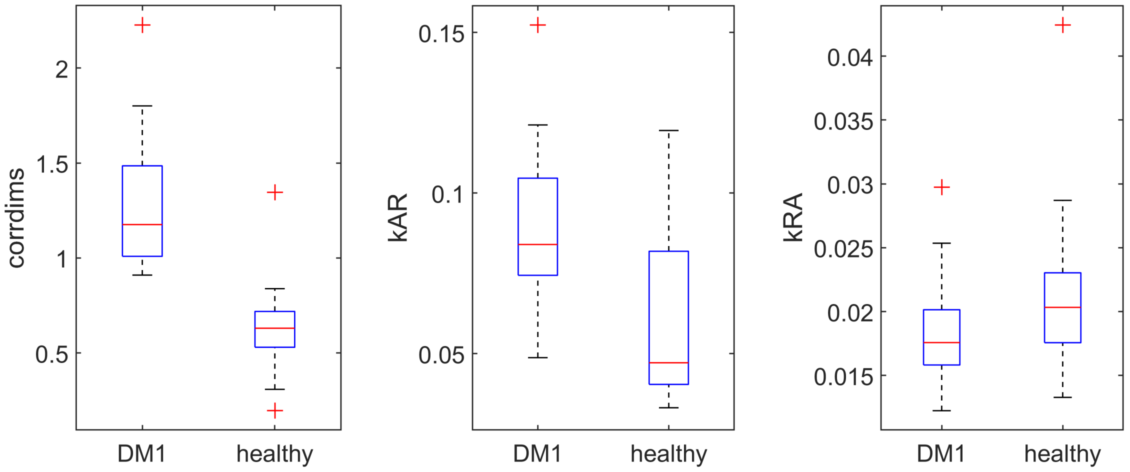


Figure 3: Re-analysis of the differences between the DM1 and healthy group for our three complexity indicators on a sub-sample which was chosen in such a way that the DM1 group matched the healthy group with respect to age and gender.

Table 1: Binary logistic regressions on our downsampled data where the 19 DM1 patients were matched to healthy individuals based on sex and age. Logistic regressions compare the analysis with the full set of traditional analysis (model 1) and the extension of this model with either the correlation dimension (model 2), kAR (model 3), and kRA (model 4). Low p-values are indicated with $^o$ (p<0.1), * (p<0.05), or ** (p<0.001). The Aikake Information criterion and Bayesian Information Criterion are calculated in rows AIC and BIC.

| | Model 1 | Model 2 | Model 3 | Model 4 |
|---|---|---|---|---|
| Avg | 3 (0.50 - 17.8) | 5600 (0.011 - 2.94x$10^6$) | 4.3 (0.48 - 39.2) | 3.1 (0.5 - 18.8) |
| CoV | 1.7 (0.19 - 15.1) | 3.6 (0.039 - 334) | 3.2 (0.22 - 46) | 3.0 (0.19 - 47.4) |
| AC | 17 (1.2 - 262)* | 9.8 (0.028 - 3.49x$10^3$) | 20 (0.22 - 46)$^o$ | 10 (0.66 - 162) |
| Cd | - | 0.0019 (0 - 1.19)$^o$ | - | - |
| kAR | - | - | 0.28 (0.047 - 1.68) | - |
| kRA | - | - | - | 1.8 (0.33 - 9.59) |
| AIC | 30.78 | 19.26 | 30.27 | 32.18 |
| BIC | 37.34 | 27.45 | 38.46 | 40.09 |

# 4 Correlation dimension - additional analysis

In order to understand the behaviour of the correlation dimension analysis, we applied the metric to various systems. In this way we can (1) verify that the code works as it should by comparing it to known systems, (2) train our intuition to help with the interpretation of our analysis, and (3) perhaps inspire others to ask more questions and expand on the analysis done here.

## 4.1 Hénon map

The Hénon map is a classical example of a two-dimensional chaotic attractor. Here, we apply the correlation dimension analysis to 10,000 points of the Hénon map. The lines in Fig. 9(a) are all straight and for dimensions larger than 1 all lines are parallel (Fig. 9(b)). Thus for the Hénon map, calculating the correlation dimension is largely independent of r (as long as it is sufficiently low) and d (as long as it is sufficiently high).

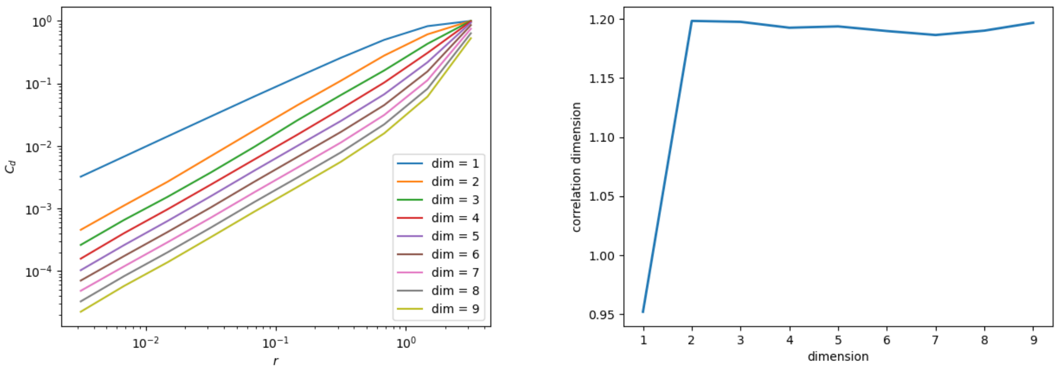


(a) Correlation integrals (b) Correlation dimension

Figure 4: Correlation dimension analysis for the Hénon map. (a) Correlation integrals for dimension 1 up to 10. (b) The correlation dimension calculated as the slopes of the lines of figure (a), in their linear regime.

## 4.2 Noisy data

We assume that our data cannot be described by a low-dimensional deterministic chaotic attractor like the Hénon map. Firstly because we can not be certain that activity patterns are governed by chaotic dynamics, secondly because our data is noisy. The algorithm for calculating correlations dimensions can easily be applied to noisy time series, but the theoretical meaning of the concepts disappears and the results are more dependable on the parameter values (Fig. 5).

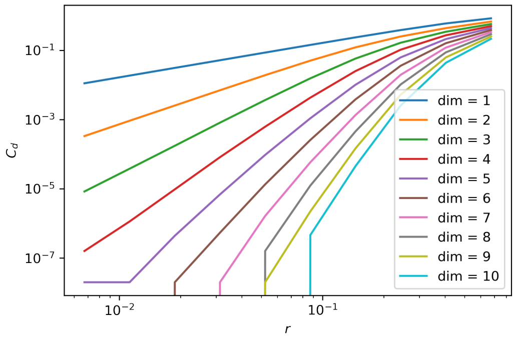


Figure 5: Correlation integrals for dimensions 1 to 10 for a red noise signal with a length of 10,000 points. The lines are not linear and their slope keeps increasing for increasing dimensions.

In our study, we applied the correlation dimension even though we are aware that the theoretical meaning is lost. However, we still believe the correlation dimension analysis is able to capture unique and clinically useful information from the signal. When comparing different signals, we ensure that the parameter values are the same and we rerun all our analyses with multiple parameter sets and we require our main results to be independent of out choices. In this way, we ensure that we are not 'cherry picking' our parameter values.

# 5 Correlation dimension - Parameters and sensitivity tests

## 5.1 Distance $r$ and dimension $d$

As explained above, for deterministic low-dimensional chaotic attractors it should be possible to choose a range of $r$ between which the lines in the $r$-$C_d$ plot are linear. Any range of $r$ within this linear regime will yield the same slope. If $r$ is chosen too low, the signal can become dominated by noise as low values of $r$ yield a low number of neighbors and thus weaker statistics. If $r$ is chosen too high, all points are neighbors from each other, leading to inaccurate estimates.

In past studies, the range of $r$ has been chosen in several different ways. Regardless of the specific choice, it has become clear than in order to compare signals to each other, as we are doing here, parameter values should be the same for the different signals [1, 2]. Therefore, various methods for determining the range of $r$ based on each individuals signal (see for example [3]) are not applicable in our case. Instead, we choose to pick the most simple heuristic, namely to let $r$ range from 0.03 to 0.3 times the standard deviation of the signal (a range around the well-studied value of 0.2 that is used for other time delay-embedding methods, see [1]), and to test the sensitivity of our choice. Our main reason for this is that we are not necessarily dealing with low dimensional deterministic chaotic attractors here and thus most formal methods are not applicable to our data. As there are several methods to pick a range of $r$, that all yield different numbers, our choice is somewhat arbitrary. If it is arbitrary, we like to keep it simple. Nevertheless, to ensure that our results do not depend on our arbitrary choice, we run several additional tests with different ranges of r. We require our results to remain the same for deviations away from the chosen range.

The choice for $d$ was more difficult, as in the example above, the slopes are still increasing for increasing values of $d$, and thus the correlation dimension is affected by this choice. We found that for most healthy individuals, the correlation dimension had converged for $d = 6$ (Fig. and see

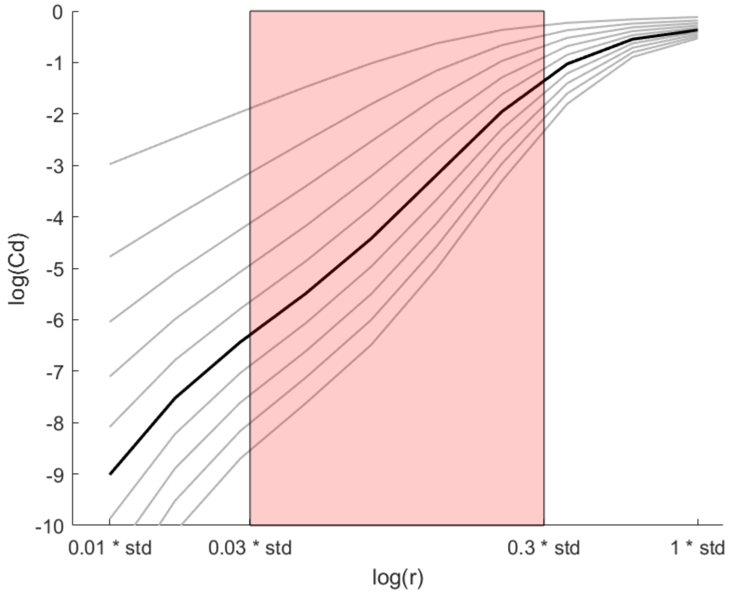


Figure 6: Correlation integrals for dimensions 1 (top line) to 10 (bottom line) for a DM1 patient. The black line is for D=6. The shaded area represents values of r that capture 0.03 to 0.3 times the standard deviation of the time series. The slope of the black line in this range is what we use as the correlation dimension for this individual.

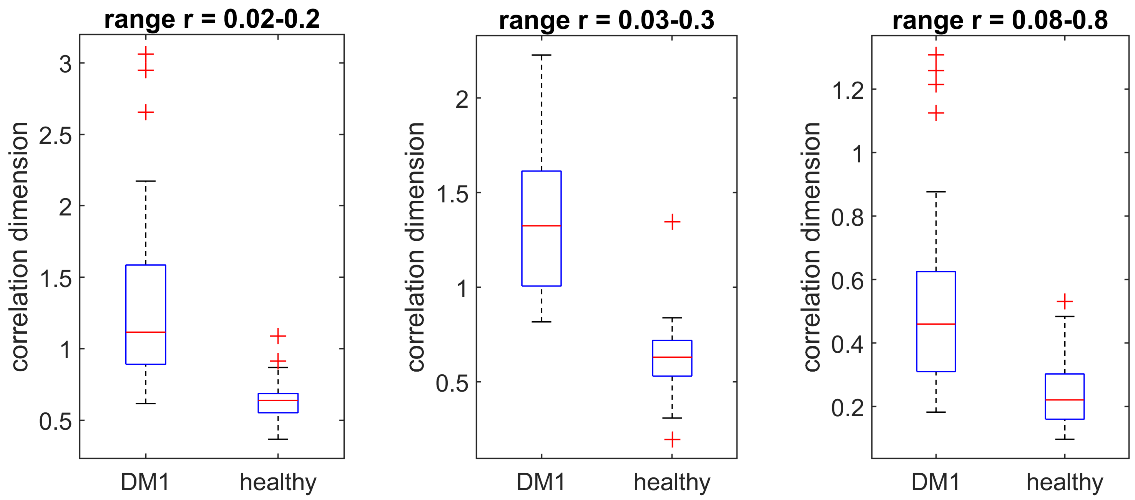


Figure 7: Comparison of the correlation dimension analysis for different choices for the range of r that was chocen: 0.02-0.2, 0.03-0.3 (default) and 0.08-0.8. The lower the range is, the higher the correlation dimension for both groups. Qualitatively the results remain unchanged.

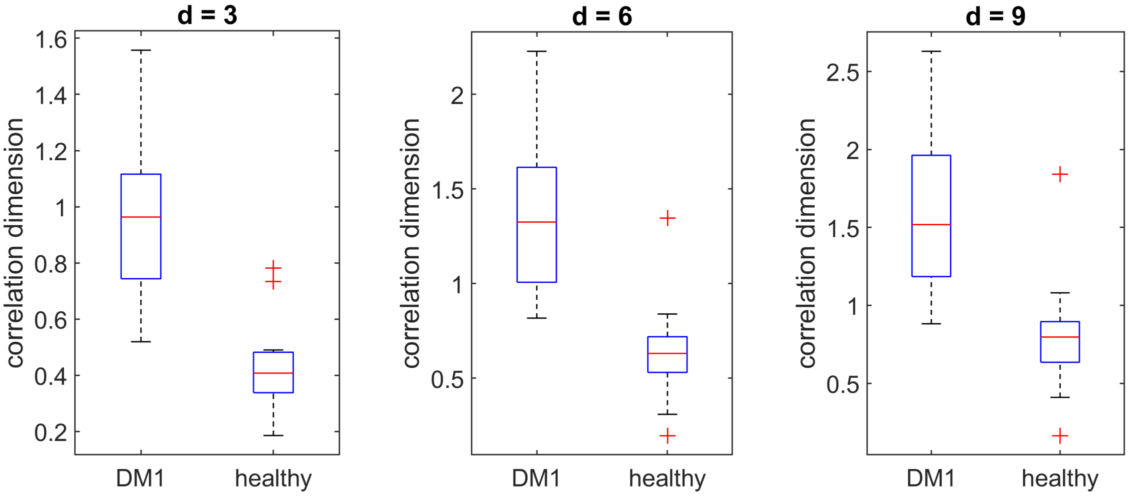


Figure 8: Comparison of the correlation dimension analysis for different choices for the embedding dimension parameter $d$: 3, 6 (default) and 9. Correlations dimensions are higher for both groups as $d$ increases, but qualitatively the results remain unchanged.

section 6.2 for a possible meaning of this finding). For the DM1 patients the correlation dimension had not converged yet, but we saw no sign of convergence for values of $d$ up to 40, so we accepted a dependency on this decision. Nevertheless, just like our choice of $r$, we require our results to be robust for deviations away from the chosen values. Different ranges of $r$ and different values of $d$ do quantitatively yield different values for the correlation dimension, but qualitatively they do not change our results that healthy individuals have a lower correlation dimension than DM1 patients (Fig. 8)

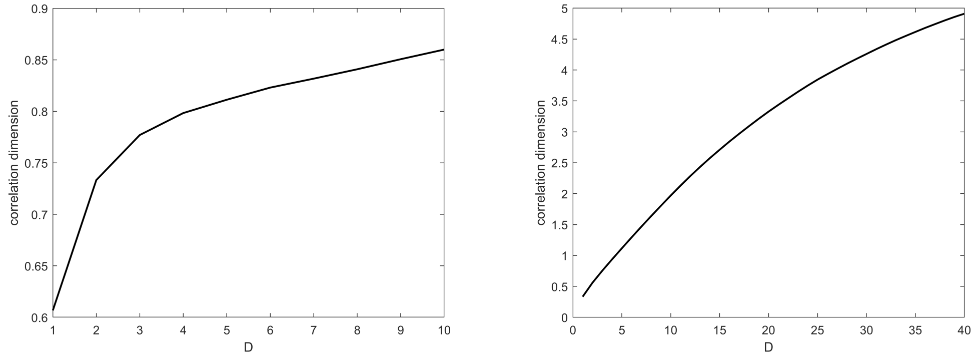


(a) Healthy (b) DM1

Figure 9: Two examples of a correlation dimension analysis for different dimensions (D), showing convergence in correlation dimension for the healthy individual and an increasing line for the DM1 group for dimensions up to 40.

## 5.2 Effect of time series length

Since not all time series had the same length, we re-ran our analysis where we cut off all time series after exactly one week, as to make sure all time series had equal length. Our results are mostly unaffected by this change (Fig. 10).

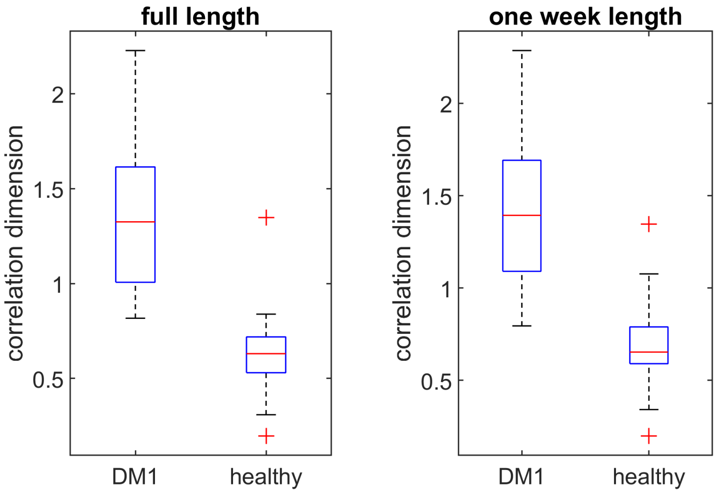


Figure 10: Comparison of the correlation dimension analysis for different choices for the embedding dimension parameter $d$: 3, 6 (default) and 9. Correlations dimensions are higher for both groups as $d$ increases, but qualitatively the results remain unchanged.

### 5.3 Different time scales

Instead of sampling at minute-to-minute intervals, we repeated our analysis with the raw data (5 second intervals) and at data resampled at the hour level. Values of $r$ and $d$ where not changed. We found that the 5 seconds data and the 1 hour data showed smaller differences between the DM1 and healthy group, but the qualitative conclusion remains unchanged (Fig. 11).

An alternative way of studying the different timescales in the system is by changing the distance of data points used in the vector for the calculating the correlation dimension. In our original study we said $X_i$ is a vector of consecutive values of the time series $x_i, x_{i+1}, ..., x_{i+d}$. Instead we could say $X_i$ is a vector of values of the time series $x_i, x_{i+1\tau}, ..., x_{i+(d-1)\tau}$. This allows us to change the value of $\tau$. This analysis reveals no qualitative changes for varying values of $\tau$ (Fig. 12).

### 5.4 Chebyshev distance

Instead of the euclidean distance, the Chebyshev (or maximum) distance can be used as well. When we rerun our analysis with the chebyshev distance, the conclusion remains unchanged (Fig. 13).

## 6 Correlation Dimension - Possible explanations

For deterministic chaotic attractors, a high correlation dimension indicates a high complexity. It is therefore perhaps surprising, that in our analysis, healthy individuals have a lower correlation dimension than the DM1 patients.

We believe that the explanation is not simply that DM1 patients have a more complex activity pattern than healthy individuals, because this explanation only holds for deterministic chaotic attractors. Furthermore, the way ‘complexity’ is defined and quantified is a topic of discussion that is beyond the scope of the present study (see for example [4] for an overview of problems and solutions of conceptualizing and quantifying complexity). Instead, we believe the algorithm picks up on a different property of the signal related to the shape and ‘chaoticness’ of the attractor. These explanations, not mutually exclusive and probably not complete, provide pointers for future work. Below, will shortly describe both explanations.

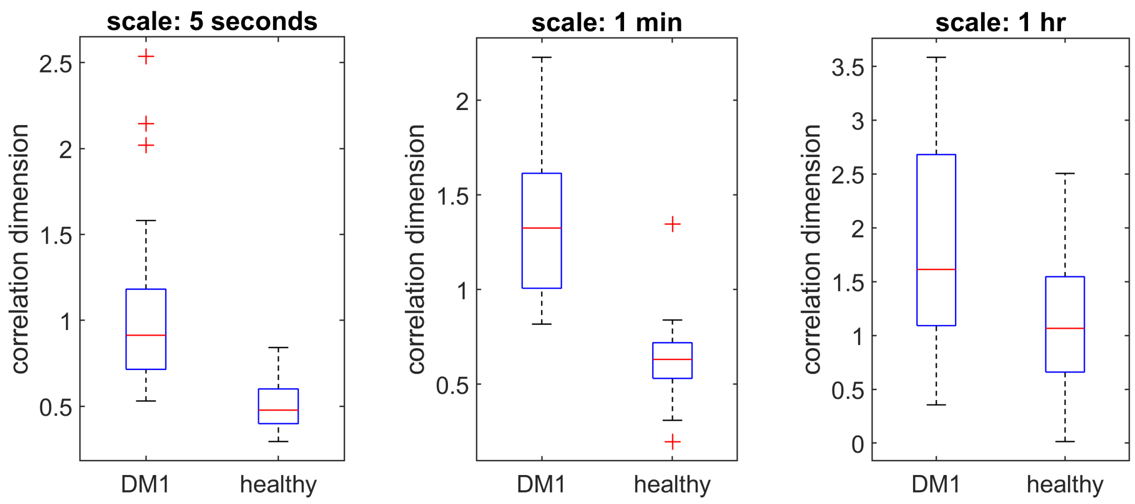


Figure 11: comparison of the correlation dimension analysis where the data is aggregated for different time scales: 5 seconds, 1 min (default), or 1 hour. The 1 minute bouts are best equipped to separate the healthy group from the DM1 group, but the other measures show the same pattern.

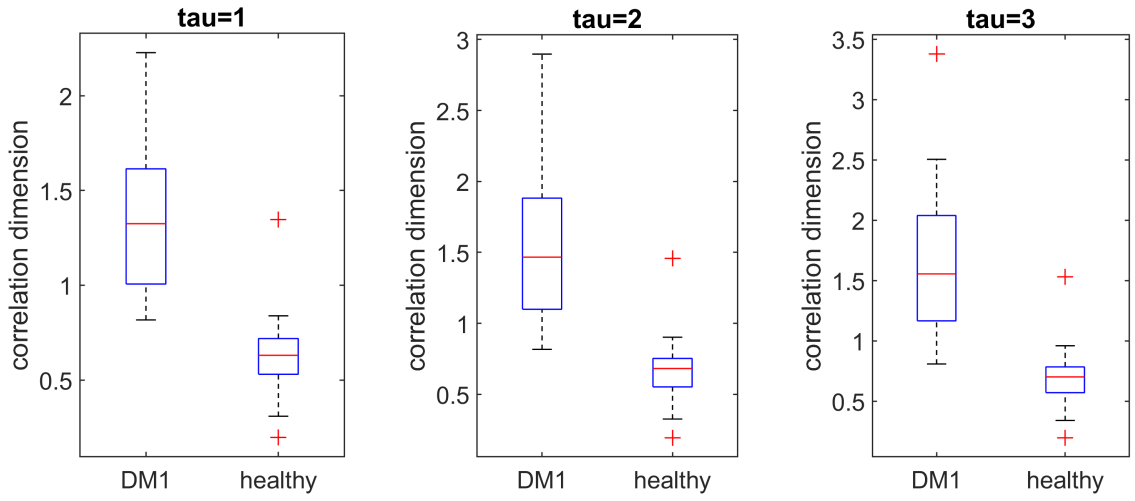


Figure 12: comparison of the correlation dimension analysis where the data is aggregated for different time scales: 5 seconds, 1 min (default), or 1 hour. The 1 minute bouts are best equipped to separate the healthy group from the DM1 group, but the other measures show the same pattern.

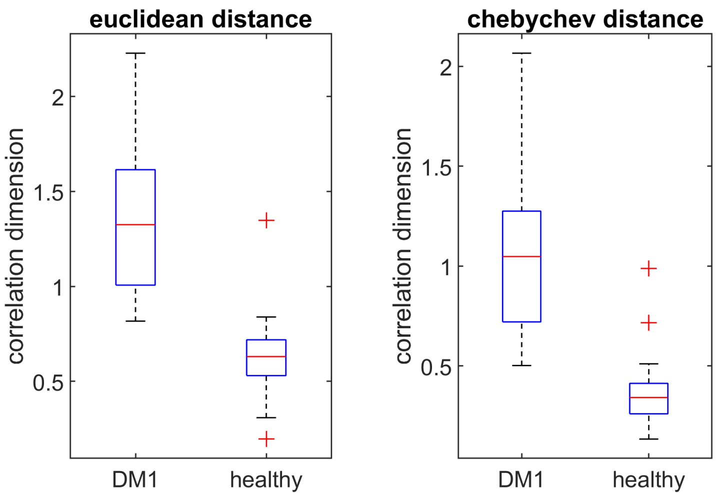


Figure 13: comparison of the correlation dimension analysis with the euclidean distance (as used in all other analysis) and the chebyshev distance as an alternative distance metric. Results are highly similar.

## 6.1 Healthy activity patterns are more extended

We can plot the time series against shifted versions of themselves to obtain a time delayed embedding (as done in figure 1 C-D in the main text). This embedding can provide some intuition about the underlying dynamics. We performed this step for all individuals in our study. In the time delayed embeddings for the healthy individuals there seem to be some regions that the system rarely visits that are not present for DM1 patients (Fig. 14). These are excursions of high activity. These excursions affect the standard deviation of the time series. As the parameter r is chosen as a fraction of the standard deviation of the time series, r has higher values for the healthy subjects than for the DM1 patients. Therefore, the healthy individuals start with a higher number of neighbors than the DM1 patients. As all correlation integral lines max out at 1 (r is so high that all points are considered neighbors), a higher number of neighbors for a certain r leads to a lower increase of number of neighbors for increasing r and this **increase** is what we calculate as the correlation dimension.

However, the higher standard deviation for healthy subjects compared to DM1 patients cannot be the full explanation, as in our analysis we correct for the coefficient of variation and find that the correlation dimension captures information that is different from the standard deviation.

## 6.2 Healthy activity is more chaotic, DM1 activity is more random

Another possible explanation of the difference that we find is that the activity patterns of the healthy individuals more closely resemble dynamics of a low dimension chaotic attractor than that of the DM1 patients. For most healthy individuals, the correlation integrals plots look more like the Hénon map example than to the red noise example (comparing Fig. 15(a) to Figs. 9 and 5). In contrast, the correlation integral lines for DM1 patients keep increasing for increasing values of the dimension, making them more similar to the red noise example than to the Hénon map (comparing Fig. 15(b) to Figs. 9, 5).

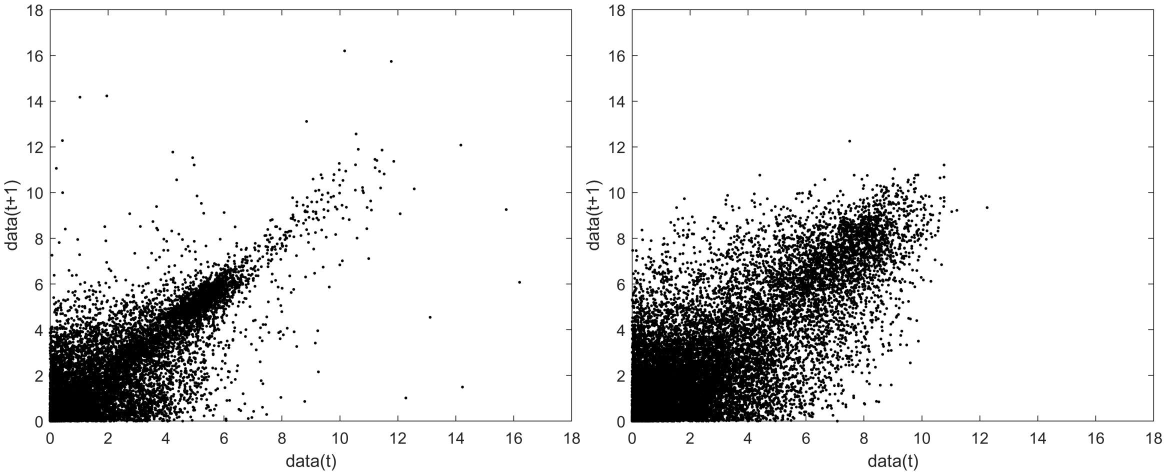


(a) healthy

(b) DM1

Figure 14: Typical time delay embeddings (lag 1) for a healthy individual and a DM1 patient. Both time series are scaled to have a standard deviation of 1. The figures indicate a more extended figure and less filled in shape for the healthy individual than for the DM1 patient. Furthermore, the healthy individual demonstrates larger excursions, despite having the same standard deviation as the DM1 patient.

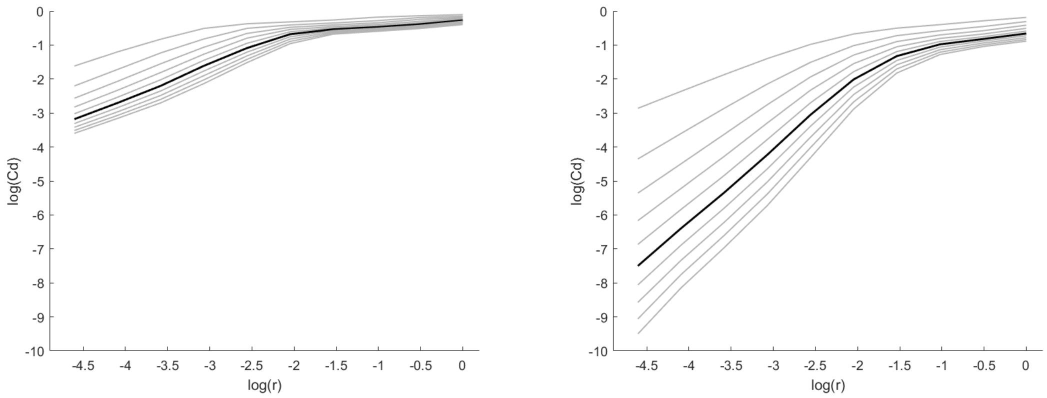


(a) healthy

(b) DM1

Figure 15: Typical correlation integral plots for a healthy individual and a DM1 patient for increasing dimensions from 1 to 10 and a range of r from 0.01 to 0.8 times the standard deviation of the data.

# 7 Fragmentation sensitivity analysis - Epoch length

To assess the sensitivity of the fragmentation measures to changes in epoch length, we varied this parameter over a range of values, re-calculated $k_{AR}$ or $k_{RA}$ for each, and the constructed a Spearman correlation. The default parameters were as follows:

- Epoch length: 1 minute
- Rest-Activity threshold: 0.2 $g$
- LOWESS bandwidth: 0.30
- Constant region definition: varying no more than 1 SD from the corresponding $pAR(t)$ or $pRA(t)$ curve

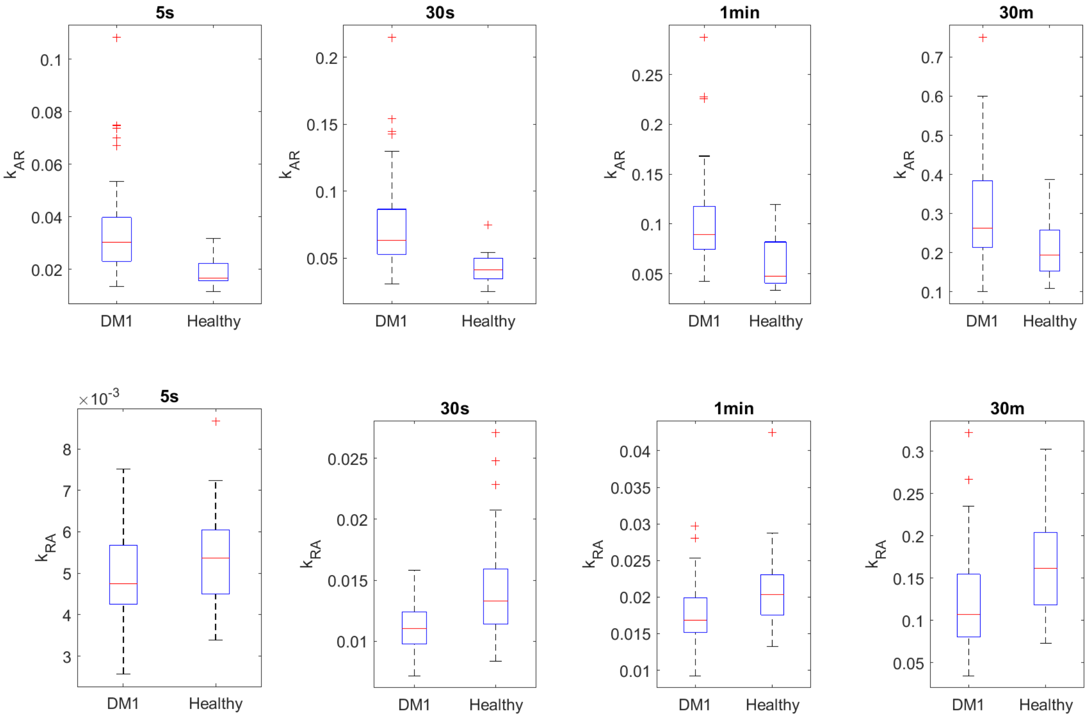


Figure 16: Box plots showing comparison of fragmentation indices $k_{AR}$ (top row) and $k_{RA}$ (bottom row) for varying epoch lengths (5 seconds, 30 seconds, 1 minute (default), and 30 minutes).

Table 2 shows the Spearman correlation matrix for $k_{AR}$ or $k_{RA}$ calculated with epoch lengths of 5 seconds, 30 seconds, 1 minute, and 30 minutes. The correlations for $k_{AR}$ are in the lower triangle and those for $k_{RA}$ are shown in the upper triangle of the table. With the exception of the 5 second-30 minute correlation ($p =$ .181), all correlations were significant across epoch lengths. These correlations were generally stronger for $k_{AR}$ than for $k_{RA}$.

Table 2: Supplementary comparison of epoch length for fragmentation analysis. Lower triangle is $k_{AR}$ and upper triangle is $k_{RA}$. Values are Spearman's $\rho$ ($p$-value).

| | **5 sec** | **30 sec** | **1 min** | **30 min** |
|---|---|---|---|---|
| **5 sec** | – | 0.38 (.001) | 0.25 (.025) | 0.15 (.181) |
| **30 sec** | 0.76 (<.001) | – | 0.68 (<.001) | 0.33 (.003) |
| **1 min** | 0.64 (<.001) | 0.74 (<.001) | – | 0.27 (.016) |
| **30 min** | 0.45 (<.001) | 0.47 (<.001) | 0.35 (.002) | – |

# References


[1] Steven M Pincus, Igor M Gladstone, and Richard A Ehrenkranz. A regularity statistic for medical data analysis. *Journal of clinical monitoring*, 7(4):335–345, 1991.

[2] Sebastian Zurek, Przemyslaw Guzik, Sebastian Pawlak, Marcin Kosmider, and Jaroslaw Piskorski. On the relation between correlation dimension, approximate entropy and sample entropy parameters, and a fast algorithm for their calculation. *Physica A: Statistical Mechanics and its Applications*, 391(24):6601–6610, 2012.

[3] Alexey Mekler. Calculation of eeg correlation dimension: Large massifs of experimental data. *Computer Methods and Programs in Biomedicine*, 92(1):154–160, 2008.

[4] Peter Grassberger. Randomness, information, and complexity. *arXiv preprint arXiv:1208.3459*, 2012.
