# Supplementary material for: Signals of complexity and fragmentation in accelerometer data
Source: PLoS One. 2025 Jul 9;20(7):e0326522. doi: 10.1371/journal.pone.0326522 (PMC12240345; doi:10.1371/journal.pone.0326522)
Supplement: Attachment — Submitted filename: Response to Reviewers.pdf [file pone.0326522.s002.pdf]

1. **The authors are advised to provide more details on parameter selection in the methods section and discuss how these choices affect the results.**

In our study, we actually spent a lot of time on parameter selection and sensitivity analysis of the parameters. We realize this information was "hidden away" from the reader a bit, thanks for pointing this out. We now highlight this aspect by using a separate section header for 'parameter selection' in our methods and we have rewritten the section to highlight its importance (L 166-194).

We have added some text to the results section of our manuscript about parameter selection and show our results are insensitive to changes in our parameters (L 290-292).

2. **Further discussion on the finding that a lower correlation dimension may imply a more predictable pattern is recommended.**

The link between a lower correlation dimension and a more predictable pattern is actually not a finding by us, but a known property of the correlation dimension. We have now placed proper references to ensure that we credit this knowledge to the correct scholars (L 315).

3. **The potential clinical implications of these findings should be explored.**

We have added a paragraph to our discussion where we highlight the limitations of our studies and the potential for future research. We hope this future research also links to the clinical implications of our work. Direct clinical implications are not the key focus of our study, but we do agree there is potential and we hope this paragraph also clarifies this to the reader (L 360-373, L 420-423).

I recently had the opportunity to read your manuscript and found it intriguing, particularly from a complex systems perspective. However, I have some major concerns regarding certain technical and methodological aspects. My major concerns are outlined below.
Thank you for reading and assessing our manuscript!

The sample size used for the main analysis (cross-sectional, as far as I understand from the manuscript) is very small. Additionally, one group was observed for 2 weeks, while the other group was observed for only 1 week. The sample sizes of the two groups are also notably imbalanced. The U statistic from the Mann-Whitney test is calculated based on the ranks of the combined groups. When one group is much larger, the ranks of the smaller group are spread thinly across the larger group's ranks, which can bias the results. Finally, no power analysis was performed. While I understand that this was a convenience sample used to test the research questions, these limitations raise concerns about the reliability of the current findings.

We agree with the reviewer that a larger sample size would provide a higher accuracy of the results. We would like to stress that this was a highly explorative study. Therefore, we did not have a clear hypothesis with regard to the effect size, and therefore we could not conduct a power analysis a priori.

To investigate the reviewer's suggestion, we performed a power analysis after our analysis, using our found effect size, and we obtained n=8 for a power of 0.95 under the assumption of normality, which would lead to ~8*1.2 = 10 individuals (20% increase and rounded up to be conservative) given the non-parametric situation we are dealing with. Our sample size is well above this number. To further gauge the power of our non-parametric analysis, we approximate the power of our test for the correlation dimension by monte carlo simulation. Here, for sample sizes from 1 up to 15, we take 1000 samples for each sample size (equal samples from both groups), where we sample from our original population, perform the mann-whitney test and compute the p-value. We then calculate the power as the fraction of simulations that lead to a significant outcome.

With a p-value of 5% we find the following graph:

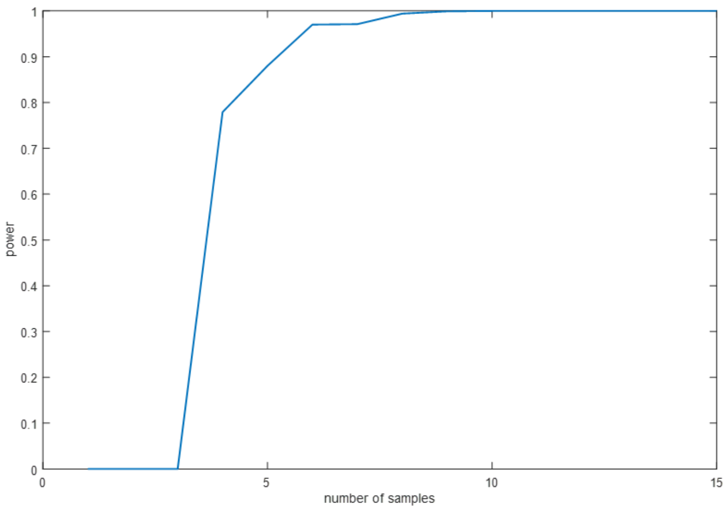

And with a p-value of 0.01 we find the following graph:

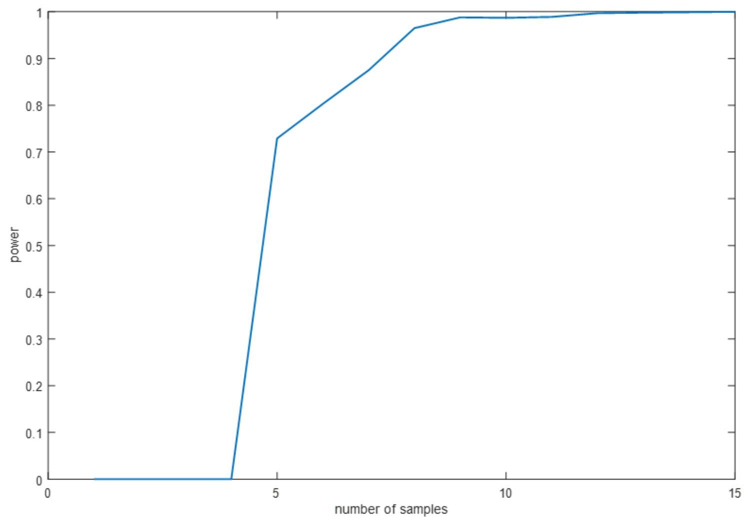

And finally, with a p-value of 0.01 and unequal sample sizes where the sample size of the DM1 group is 3 times as high as the healthy group (as is the case in our main analysis):

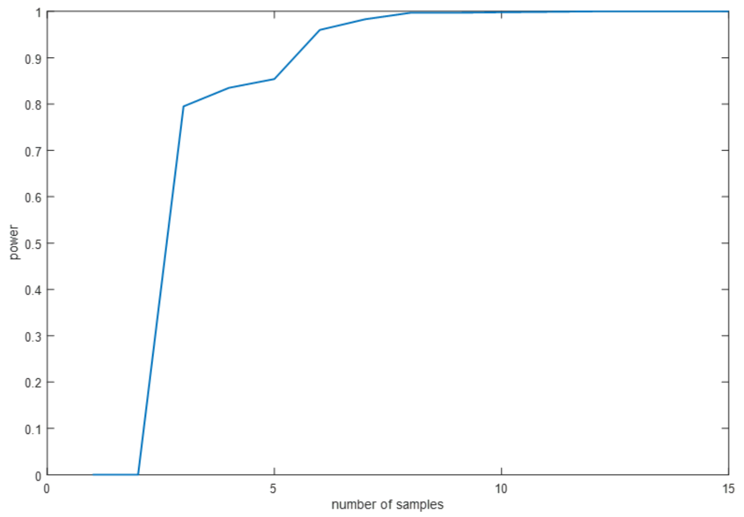

Note for this last image that the 'number of samples' refer to the number of samples for the healthy group, so the number 5 means 5 healthy individuals and 15 DM1 individuals.

In both cases, we see that from a sample size of 10 onward, the power is higher than 99%. This gives us confidence that the effect we found is big enough that our relatively small sample size picks up on it. We have now added the first one of these graphs to our supplementary materials.

Furthermore, we'd like to stress that after our main analysis, we asked ourselves the same question that the reviewer has asked us. Therefore we repeated our analysis with a subset of individuals from the DM1 group, to match the sample of the healthy individuals with respect to age and gender. We presented this analysis in our supplementary materials and described it in our results section. The

reviewer's comments made us realize that we should highlight this analysis more and in our new manuscript we give this analysis a more prominent role in our discussion section (L 363-366).

That said, the reviewer is absolutely right that even with the addition of the power analysis and this additional matched sample analysis, our sample size will remain a weakness of our study, and we now emphasize this caveat more explicitly in our discussion section (L 360-373). We hope that our work will inspire future research to explore these complexity indicators on accelerometer data on larger datasets.

Higher correlations are likely descriptive of the symptoms that characterize the DM1 group—and the broader population with myotonic dystrophy type 1—rather than being indicators capable of distinguishing between groups with known health differences. Specifically, from the protocol paper (van Engelen, 2015), I understand that this population is characterized by daytime sleepiness. Therefore, one might assume that the highly irregular signals in the DM1 group are due to this condition. On a related note, in line 370 (page 15), the authors state that "Healthy individuals had several consecutive hours of low activity every 24 hours, whereas individuals with DM1 had multiple periods of low activity scattered throughout the day and night (linking to their high active-to-rest transition probability). Whereas previous studies have chosen to remove periods of sleep [9], the complicated sleep pattern of the DM1 group did not allow us to follow this approach." The fact that the complex sleep patterns prevented the authors from removing sleep periods further supports my view that the high correlation in accelerometer data might be typical of this clinical sample, rather than indicative of a generic unhealthy population. A different comparator is needed to assess the discriminative value of the correlation measures to distinguish between groups with known health differences.

We thank the reviewer for this critical but very valid point. Indeed, we only test the complexity indicators on individuals in a 'healthy' group and in a 'DM1' group, so we should be careful with claims about general 'health' or 'disease' based on our results. We have made several textual edits to make sure we do not overstate the implications of our findings, in line with the reviewers comment (see tracked document).
