# Supplementary material for: Signals of complexity and fragmentation in accelerometer data
Source: PLoS One. 2025 Jul 9;20(7):e0326522. doi: 10.1371/journal.pone.0326522 (PMC12240345; doi:10.1371/journal.pone.0326522)
Supplement: Attachment — Submitted filename: rebuttal_05_2025.pdf [file pone.0326522.s003.pdf]

**1. As the reviewer pointed out, the control sample lasted a week less than the DM1 sample. Correlation dimension is well known to be affected by sample length. Have the authors already checked if reducing the sample size to a week affects the differences observed? If not this would be worth investigating**
we re-ran our analysis with time series that are all exactly one week of length, obtaining the following fig:

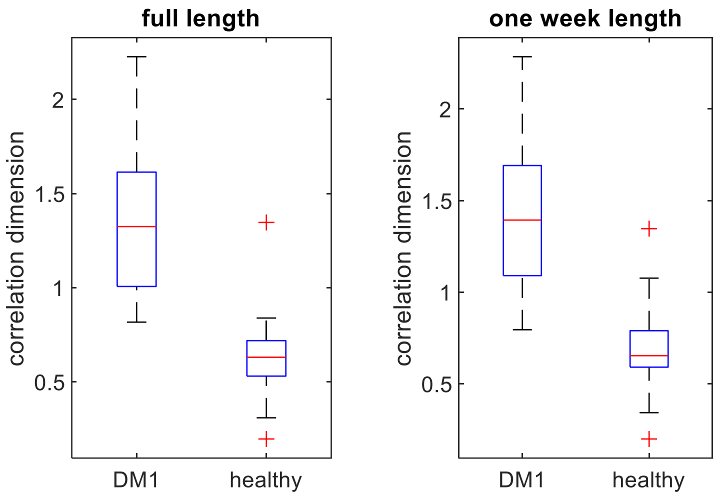


Where the left fig is our basic analysis as presented in the paper and the right fig represents the same analysis but time series that are cut off after one week. This fig indicates only minor changes when the time series are cut-off after one week.

We now added this figure to our SM, and added the following line to our methods section:

"In our main analysis we ran all analysis on the full length datasets. In our sensitivity tests, we tested whether the uneven lengths between the DM1 group and the healthy group affected our results." (L105-107)

**2. I checked the github repo to check the above, but noticed that the code used for calculating the complexity measures were not available. Could this be added to the repo?**
Done

**3. I understand that the authors interpret correlation dimension in the context of random data, but since the measure is originally developed for the analysis of signals exhibiting deterministic chaos, it would be nice to have an interpretation of what that is. In the supplementary, the authors have considered purely deterministic signals as well for their analysis. While it is generally true that regularity implies lower dimension and randomness or chaos implies higher dimensions, Correlation dimension is in essence a measure of fractality. I would recommend adding a few lines describing what exactly it is measuring in the paper, especially since a lot of the readers may be non-specialists.**
We added the following line: "The correlation dimension is linked to the `fractal dimension' of the dynamical system, i.e. it describes the patterns and shape of the signal. It does so, by calculating patterns of repeated sequences in the data." (L40-42)

**4. Choice of tau=1: While in theory Taken's theorem guarantees that any value of delay time, tau, will work, for real datasets it is recommended that a delay time where the**

**correlation (linear or otherwise) decays is chosen. Common thresholds are where the autocorrelation falls to 1/e or first minimum of the mutual information etc. It would be worthwhile to consider adding a sensitivity analysis that looks into these.**
This relates to the relevant time scale of the dynamics in the system. In our manuscript, we opted for a different approach and tested for the sensitivity of coarse graining over a larger range and found a robust signal with respect to changes of course graining scale (Supplementary fig. 10).

Nevertheless, it is an interesting suggestion to also test the sensitivity of tau directly, which we did in the figure below, which is now also added to the supplementary materials:

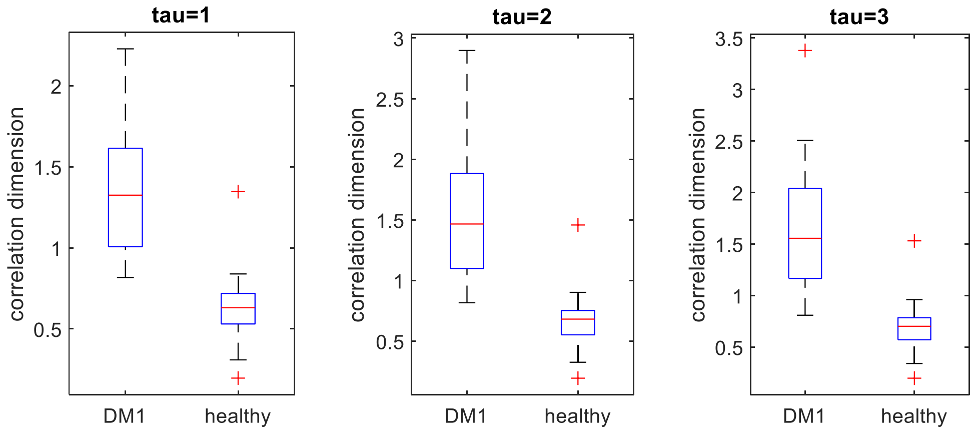


And we added the following sentence to the methods:

"and the time interval between the data points used in *X* (we call this time interval $\tau$)" (L 191)

**5. Choice of dimension: The authors chose dimension of 6 as far as I understand. But I don't understand Figure 6. Is this the dimension that was obtained at dimension 6? This seems unlikely, since there was no saturation observed for DM1, according to the authors. Maybe it would be worthwhile to add average D2 at every dimension for the healthy and diseased datasets in the main text/supplementary. In addition, if D2(1/2), i.e. correlation dimension at dimension 1 or 2, was chosen for the analysis, this may not be the best choice, since at this dimension the full complexity of the embedded space will not be captured by the measure.**
This figure is indeed made with D=6. Sensitivity to this choice was tested (supplementary section 5.1).

In a way, supplementary fig 8 (sensitivity to choice of D) gives some points of the graph that the editor describes here, i.e. boxplots of D2 for different dimensions.

For illustrative purposes, we created D vs Cd plots for 2 individuals (one from the healthy group and 1 from the DM1 group) as a showcase to illustrate the typical difference between both groups:

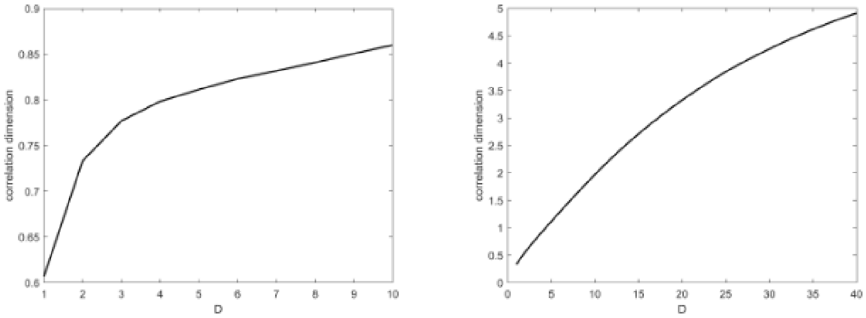


(a) Healthy (b) DM1

**6. Choice of threshold for transition probability analysis. Its set currently via visual inspection. Is this number close to a number that can be objectively defined? n times mean/median/standard deviation. What would be a suitable recommendation for future researchers looking to repeat this analysis for their datasets?**
The threshold was indeed set by visual inspection. However, it was found that the values of $k_{AR}$ and $k_{RA}$ are quite robust to selection of rest-activity threshold. These parameters correlated highly ($\rho \geq 0.93$) for all values calculated from cut-offs ranging from 0 counts to the $10^{th}$ percentile (in increments of 2.5 percentile scores). We suggest future researchers choose values that clearly exceed the baseline noise level of the activity data, given the sampling frequency and quality of the raw data.

We added the following line: “While kAR and kRA were found to be relatively insensitive to the selected activity threshold [9], this cut-off value exceeded the baseline noise level of our raw accelerometer data.” (L 208-210)

**7. Saturation curves for example datasets: In the supplementary data, the authors provide C(R) vs R curves for the data. It would make sense to add D2(M) vs M curves as well, i.e. Correlation dimension vs dimension curves. This would make the saturation observed in the healthy controls evident.**
Done in the new version (see point 5 of this letter and Suppl Fig. 9).

**8. Figure 11 in supplementary will be clearer if plotted using points alone. The lines obscure the structure.**
Done
